# Environmental coherence framework for multi-sensor remote sensing: water hyacinth assessment in Lake Tana

**DOI:** 10.1038/s41598-026-46912-0

**Published:** 2026-04-30

**Authors:** Mohamed Rami Mahmoud, Luis A. Garcia, Ahmed Abd Elhamid, Mostafa Aboelkhear

**Affiliations:** 1 National Water Research Center, Ministry of Water Resources & Irrigation, Cairo, Egypt; 2 Civil and Environmental Engineering Department, University of Vermont, Burlington, VT 05405 USA; 3 Department of Physics and Astronomy (DIFA), University of Bologna, Bologna, Italy

**Keywords:** Water hyacinth, Remote sensing, Environmental coherence, Multi-sensor fusion, Lake Tana, Google Earth Engine, Invasive species monitoring, Blue Nile basin, Climate sciences, Ecology, Ecology, Environmental sciences, Hydrology, Natural hazards, Water resources

## Abstract

Invasive water hyacinth threatens Lake Tana, Ethiopia—the source of the Blue Nile. This study presents the first comprehensive 11-year (2013–2024) remote sensing assessment of the water hyacinth invasion dynamics. It introduces a novel environmental coherence framework—grounded in established indirect validation paradigms—to evaluate 11 multisensor algorithms (using Landsat 8/9, Sentinel-1, and Sentinel-2 data) against hydro-meteorological drivers, providing an ecologically grounded validation alternative when systematic field surveys are impractical. This environmental coherence framework identified Sentinel-2 NDVI and NDVI + FAI as the best indicators, achieving the highest relative environmental coherence scores among the 11 indicators tested. The time series generated by this framework follows a complex ‘boom-bust’ invasion cycle, with a peak phase (2018–2019), a subsequent decline, and a recent resurgence. Our findings establish a replicable, Google Earth Engine based workflow for monitoring aquatic invasions in data-scarce tropical regions and provide critical, data-driven insights for targeted environmental management.

## Introduction

Invasive aquatic plants constitute one of the most significant threats to freshwater ecosystems globally, with water hyacinth (*Eichhornia crassipes*) recognized as the world’s most problematic aquatic weed due to its rapid proliferation and severe ecological, economic, and social impacts^1,2^. In tropical and subtropical freshwater systems, uncontrolled water hyacinth infestations obstruct navigation, degrade water quality, reduce biodiversity, impair hydropower infrastructure, and compromise the livelihoods of communities dependent on fishing, irrigation, and tourism^3,4^.

Satellite remote sensing has emerged as the most viable approach for monitoring aquatic vegetation dynamics over large areas and extended time periods, particularly where field-based surveys are logistically and financially constrained^5,6^. Recent advances in cloud-computing platforms—notably Google Earth Engine (GEE)—and the growing availability of free, analysis-ready imagery from Landsat 8/9, Sentinel-2, and Sentinel-1 have enabled multi-sensor, multi-temporal monitoring workflows that were previously impractical^7^. However, significant methodological gaps remain. Most existing studies rely on a single optical sensor, cover short time spans (1–3 years), and employ limited spectral indices. Persistent cloud cover in tropical regions frequently disrupts optical time series, and radar data are rarely integrated into these time series. Furthermore, the absence of standardized validation frameworks—particularly for data-scarce regions where field-based ground truth is impractical—limits the confidence and comparability of reported results.

This study addresses these gaps by presenting the first comprehensive 11-year (2013–2024) remote sensing assessment of water hyacinth invasion dynamics in Lake Tana, Ethiopia. Specifically, this study: (a) develops a multi-sensor workflow integrating Landsat 8/9, Sentinel-2, and Sentinel-1 to produce consistent monthly areal estimates of water hyacinth extent; (b) evaluates eleven spectral index–sensor combinations using a novel environmental coherence framework grounded in established indirect validation paradigms^8^, and (c) characterizes the full temporal trajectory of the invasion, including a previously undocumented boom-bust cycle. The entire workflow is implemented in Google Earth Engine, and all code is publicly available to facilitate replication and adaptation to other data-scarce tropical water bodies.

## Study area

**Fig. 1:**
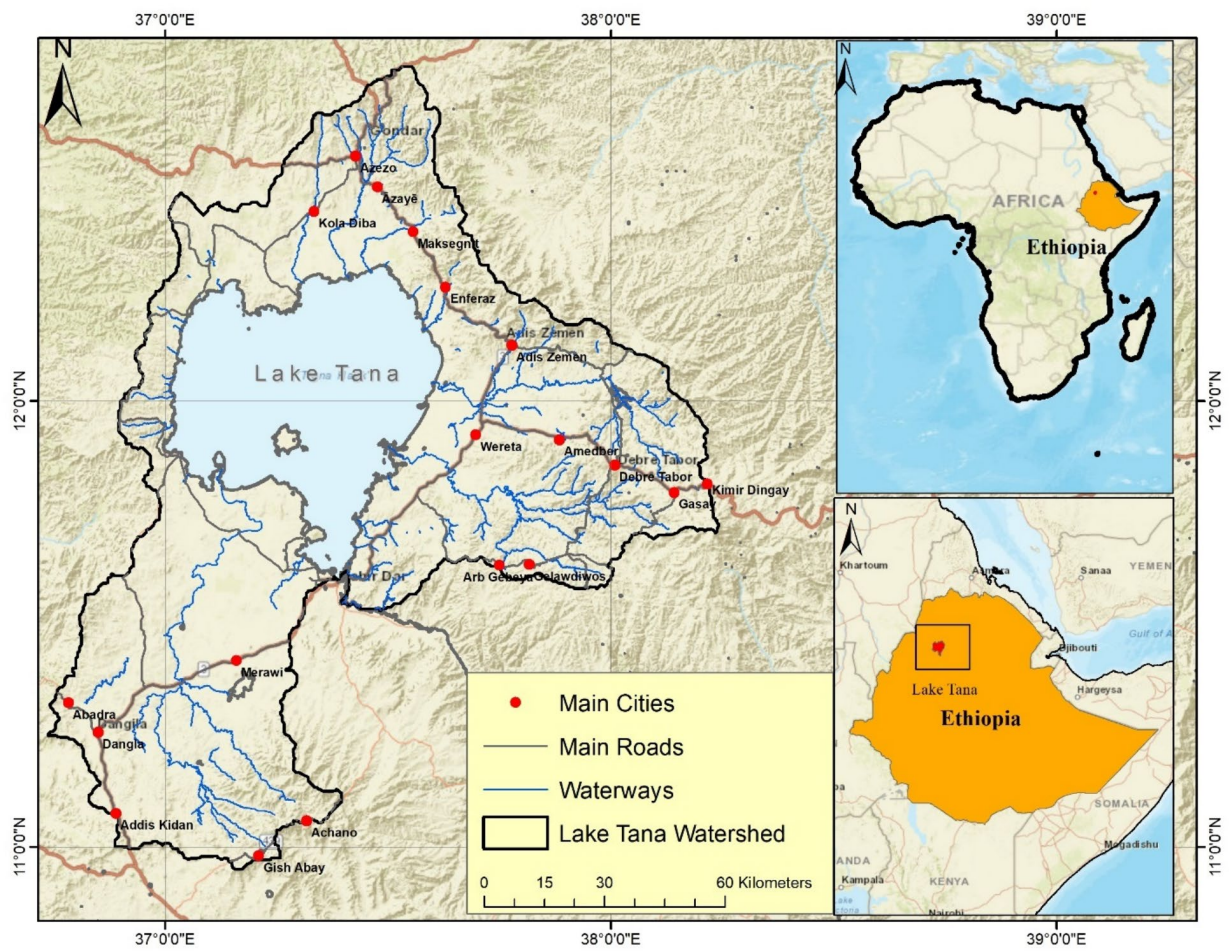
Overview of Lake Tana with its adjacent floodplain wetlands.

Lake Tana is situated in the northwestern highlands of Ethiopia (approximately 12°N, 37.3°E) at an elevation of 1786 m above sea level (Fig. 1). With a surface area of approximately 3060 km^2^, it is the largest lake in Ethiopia and the source of the Blue Nile River—a waterway of immense regional and transboundary significance. The lake has a roughly elliptical shape, a maximum depth of 14 m, a mean depth of approximately 9 m, and a total catchment area of ~ 15,000 km^2^. More than 40 rivers and streams feed the lake, with four major tributaries—the Gilgel Abay, Gumara, Ribb, and Megech rivers—contributing approximately 93% of the inflow. The lake’s total volume is approximately 28.4 billion cubic meters^9,10^.

**Fig. 2:**
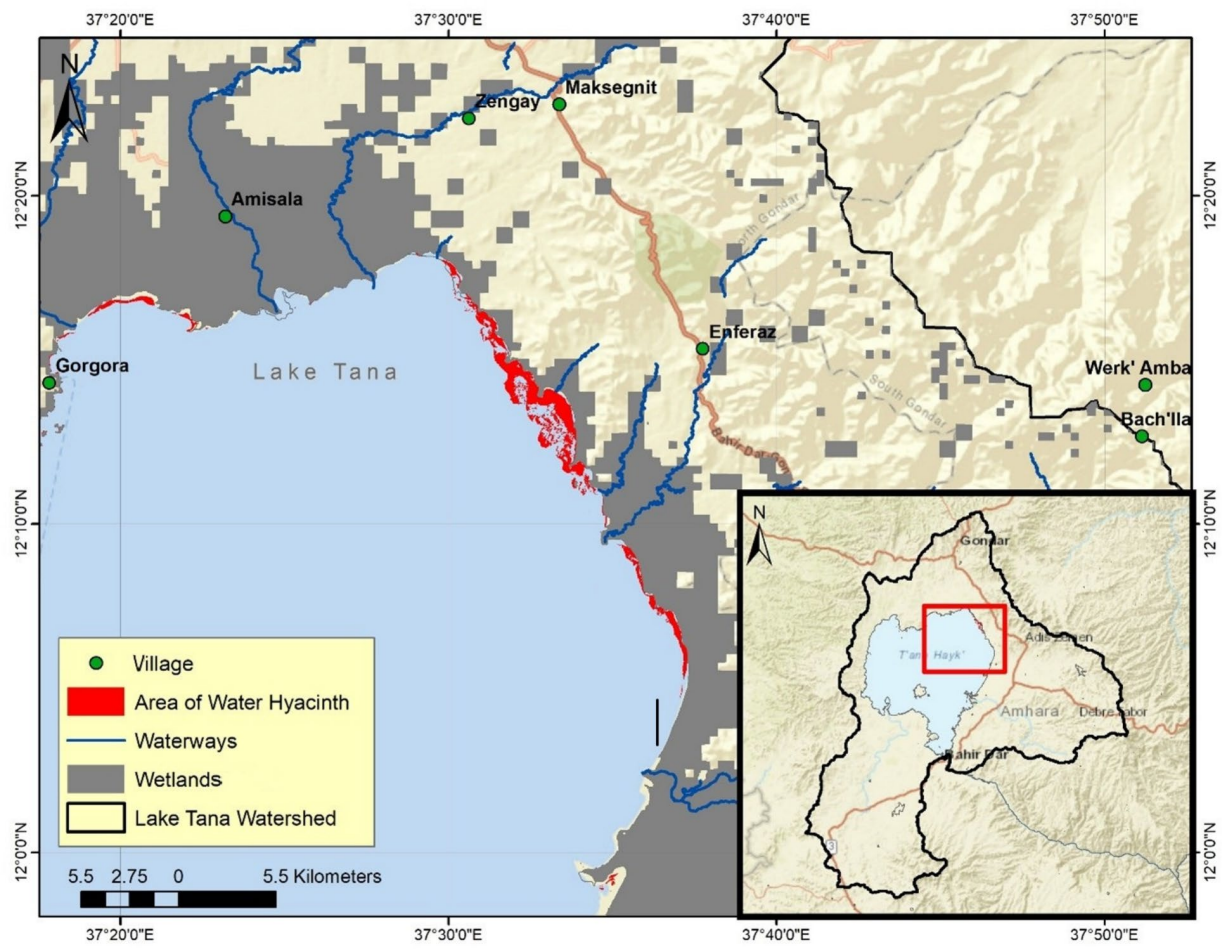
Lake Tana, December 2019, showing areas infected with water hyacinth.

The climate is governed by the movement of the intertropical convergence zone, producing a single rainy season from June to September. The surrounding floodplains (Dembiya, Fogera, and Kunzila) create extensive seasonal wetlands (~ 1600 km^2^) that provide critical habitat. The lake is listed among the top 250 globally important lake regions for biodiversity, including 18 endemic barbus fish species and at least 217 bird species^11^. In recognition of this, the Lake Tana region was designated a UNESCO Biosphere Reserve in 2015 (Fig. 2).

Since 1996, outflow from the lake has been regulated by the Chara Chara Weir for hydropower generation (Tana Beles and Tis Abay I and II hydropower stations). The lake serves multiple economic functions, including fishing, transportation, tourism, irrigation, and domestic water supply, with over 500,000 people directly or indirectly dependent on the lake and adjacent wetlands^11,12^. Water transparency is generally low due to large suspended sediment loads and bottom resuspension, with shallow areas concentrated along the northeastern shore and eastern littoral zones—the same areas where water hyacinth infestations are most severe^3,13^.

## Water hyacinth (*Eichhornia crassipes*)

Water hyacinth (*Eichhornia crassipes*) is an invasive, free-floating aquatic plant native to South America that thrives in tropical and subtropical climates. Each plant can produce thousands of seeds annually, which remain viable for decades^1^. Dense mats of the plant can double in size within two weeks, given favorable conditions, significantly impacting aquatic ecosystems^14,15^. As a result, the International Union for Conservation of Nature (IUCN) has classified water hyacinth as one of the world’s 100 worst invasive alien species due to its severe ecological and socio-economic impacts. Water hyacinth is also frequently cited among the top 10 most damaging aquatic weeds globally^16^ because, when uncontrolled, it forms thick mats that cover entire water surfaces, blocking sunlight essential to submerged plants. This leads to reduced photosynthesis and mass die-offs of native aquatic vegetation. The subsequent decomposition of plant material drastically depletes dissolved oxygen in the water, often resulting in fish kills and the collapse of aquatic biodiversity^17^. Additionally, these thick mats serve as breeding grounds for mosquitoes and snails, thereby increasing the risk of malaria and schistosomiasis in affected communities^18^.

In addition to its rapid growth and reproductive capacity, water hyacinth exerts substantial hydrological pressure on freshwater ecosystems due to its high evapotranspiration (ET) rates. Recent studies from Lake Tana estimated the mean ET of water hyacinth mats to be up to 9.05 mm/day during the dry season – more than 50% higher than surrounding open water surfaces^19^. Such additional water losses can exacerbate seasonal water scarcity in hydrologically sensitive basins, such as Lake Tana, where competing water demands for irrigation, fisheries, and ecosystem services already exist.

Water hyacinth growth is strongly influenced by climatic variables, particularly temperature, solar radiation, and nutrient availability. Optimal growth is observed in warm, humid environments with ambient temperatures between 25 and 30 °C and water temperatures ranging from 20 to 35 °C^20^, as well as in eutrophic waters, where elevated concentrations of nitrogen and phosphorus drive substantial biomass accumulation^21^. In watersheds such as the one in Lake Tana, seasonal nutrient influxes following rainfall events exacerbate this process, leading to peak infestation periods during the wet season^16^. Despite various management efforts, including the introduction of biological control agents^22^, their effectiveness has been limited. The plant’s rapid vegetative reproduction, seed longevity, and environmental plasticity enable it to recolonize treated areas and quickly regain ecological dominance^22^. These dynamics underscore the importance of early detection and integrated management strategies that consider both seasonal environmental triggers and the biological resilience of this invasive species.

Beyond ecological harm, water hyacinth significantly disrupts human activities. It clogs irrigation systems, hinders navigation and fishing, and exacerbates flooding risks by blocking waterways^23^. Control efforts often require multifaceted strategies that combine mechanical, chemical, and biological methods, though complete eradication remains extremely challenging^24^.

## Water Hyacinth in Lake Tana

Water hyacinth was first identified as an invasive species in Lake Tana in 2011 by the Amhara Regional State Environmental Bureau. A GPS-based survey conducted between October 27 and November 3, 2011, documented the presence of water hyacinth in 24 sites around the lake’s perimeter. Although the early water hyacinth infestation was determined to be primarily localized to the northern and northeastern shores, subsequent studies have confirmed that areas infested by water hyacinth have rapidly expanded^25^.

In 2014, Bahir Dar University (BDU) and the Organization for Rehabilitation and Development in Amhara (ORDA) formed a technical study team to assess the proliferation of water hyacinth in the lake. The study team conducted a reconnaissance of the lake’s eastern and northern beaches during the first week of August 2014. The study estimated that 40 km of the lakes’ 385 km of shoreline was infested with water hyacinth^26,27^. The 2014 evaluation indicated that, alongside re-infestation in previously identified regions, new locations, particularly within the Fogera floodplain, were also plagued by water hyacinth. Assuming that water hyacinth infestations persist in their expansion towards the source of the Blue Nile River. This means it will encroach on the Blue Nile River from its source, ultimately reaching the Great Ethiopian Renaissance Dam (GERD) reservoir and beyond^28^.

Between July 2013 and July 2015, Tewabe et al.^29^ conducted a study in the northeastern region of the lake, selecting six sites in the Fogera, Libokemkem, and Dembia districts according to their water hyacinth infestation levels. They estimated that 82.2 tons/ha of water hyacinth may be harvested during the dry season, while 270 tons/ha can be harvested during the rainy season.

Worqlul et al.^13^ investigated the lake’s northeastern shore using satellite data from PlantScope covering the period from August 2017 to April 2018. The goal of the study was to delineate the spatiotemporal distribution of water hyacinth and identify the principal causes driving its proliferation. Their findings indicated that water hyacinth increased from August to mid-November 2017, then declined thereafter. The decrease in infections after mid-November was attributed to a decline in lake level and a reduction in minimum nocturnal temperatures. Consequently, environmental factors are likely to be essential in shaping the spatiotemporal distribution of water hyacinth in Lake Tana.

## Remote sensing of water hyacinth

Ground-based measurements provide the most direct means of assessing aquatic weed distribution. However, they are labor-intensive, time-consuming, and spatially constrained, limiting their utility for large-scale or long-term monitoring. In contrast, remote sensing offers an efficient and cost-effective alternative, enabling broad spatial and temporal assessment of aquatic weed infestations. With this aim, various remote sensing platforms, including ground-based, airborne, UAV, and satellite systems, have been used to monitor aquatic ecosystems. Rolim et al.^30^ reviewed sensor technologies and detection algorithms with a primary focus on algal blooms. While informative, their review emphasizes algal dynamics and does not address the long-term spatiotemporal patterns of invasive macrophytes, such as water hyacinth, particularly in data-limited regions.

The following studies have used satellite sensors to monitor aquatic macrophytes; however, they lack a temporal analysis, limiting insights into seasonal or interannual dynamics. Oyama et al.^31^ used Landsat 7 imagery to classify surface cover types in Japanese lakes using combinations of FAI and NDWI indices. Although effective in distinguishing different aquatic features, their analysis did not quantify the infested area or assess the indices’ performance over time or across sensors. John and Kavya^32^ applied hyperspectral WorldView-2 imagery for the classification of aquatic vegetation in the Vembanad estuary, achieving high accuracy. However, the reliance on high-cost commercial imagery constrains scalability, and they did not explore the temporal dimension. Dube et al.^33^ demonstrated high classification accuracy using Landsat 8 for water hyacinth detection in a Zimbabwean lake for a single event. Singh et al.^6^ developed a hierarchical classification scheme in Google Earth Engine (GEE) using Random Forest modeling to identify water hyacinth at a national scale in South Africa. While the model demonstrated high accuracy, the study did not examine seasonal or multi-year trends, and the results were presented as static classifications. Gerardo & de Lima^34^ utilized Sentinel-2 indices to distinguish water hyacinth from other aquatic vegetation in the Mondego River valley. Their study was spatially explicit but lacked a temporal component and did not include radar data for periods of cloud contamination.

The following studies have monitored aquatic macrophytes with limited temporal analysis. Ongore et al.^35^ assessed the impact of macrophytes on fisheries in Lake Victoria using Sentinel-2 and Landsat imagery. While their study identified spatial hot spots, it did not provide consistent areal estimates or evaluate index-specific performance. Moreover, data gaps during cloudy seasons were not addressed. Xu et al.^36^ mapped the extent of algal blooms in Lake Taihu (China) using Sentinel-2 MSI and Landsat OLI. Although they reported valuable insights into algal bloom frequency and duration, cloud cover during key summer months severely limited image availability. The study highlighted the need for supplementary data sources during high-cloud periods. Mqingwana et al.^37^ employed GEE-based machine learning to assess biological control interventions, achieving high classification accuracy. However, the temporal scope was narrow, focusing on specific management outcomes without assessing long-term patterns of invasion. Janssens et al.^38^ applied a Naïve Bayes classifier in GEE to monitor the seasonal variation of water hyacinth in the Saigon River over three years. Although seasonal trends were observed, the limited temporal range and lack of multi-sensor comparison constrain broader ecological inference. Mouta et al.^39^ utilized UAV and Sentinel-2 data, combined with classifier fusion, to achieve high-accuracy mapping in Portugal’s Cávado River. Despite its technical robustness, the method relies on UAV data, limiting its applicability to larger or less accessible regions and not addressing long-term monitoring. Bayable et al.^40^ conducted a Random Forest classification in GEE for Lake Tana using Sentinel-2 and Landsat 8 imagery. While reporting strong classification performance, the analysis was temporally limited and lacked field validation. Additionally, cloud cover during certain months disrupted data continuity, and no radar imagery was used to mitigate this limitation.

The following studies have established empirical relationships between several environmental variables in Lake Tana and similar tropical lake systems: Abebe et al.^41^ found that in Lake Tana, spatial and temporal patterns of water hyacinth are significantly associated with seasonal climate variability and elevated evapotranspiration rates estimated using the FAO-56 Penman–Monteith method. Dersseh et al.^3^ reported a strong positive correlation (r ≈ 0.69, *P* < 0.05) between water level fluctuations and water hyacinth areal extent in Lake Tana, based on Nov-Dec observations,higher lake levels expand suitable shallow and flooded zones for water hyacinth infestation. While direct effects are complex, Mekoya et al.^42^ reported increases in ET₀ and air temperature over the past few decades in the Lake Tana sub-basin, with precipitation variability influencing the water balance. This suggests that rainfall indirectly affects water hyacinth through moisture, nutrient runoff, and dilution dynamics.

Collectively, the literature indicates that satellite remote sensing can detect and map water hyacinth; however, most studies are limited by short time spans, single seasons or sites, and restricted cross-sensor or index evaluation. Many rely on a single optical sensor or on costly hyperspectral/UAV data, which provide only snapshot classifications rather than consistent areal-time series, and seldom address persistent cloud cover (with radar data rarely used). The reported accuracy is also infrequently supported by field validation, limiting ecological inference. A stronger path forward involves a multi-year, multi-sensor workflow (e.g., harmonized Landsat/Sentinel-2 with targeted indices such as FAI/NDWI, complemented by Sentinel-1 during cloudy periods), paired with standardized areal estimation and in-situ checks, to produce management-ready time series of water hyacinth invasion dynamics as developed in this research.

## Materials and methods

All data acquisition and processing were conducted using the Google Earth Engine (GEE) cloud-computing platform, which provides access to a multi-petabyte catalog of satellite imagery and geospatial data. All processing was implemented using Python scripts in Google Earth Engine (code available at 10.5281/zenodo.17563763).

### Satellite imagery for water hyacinth detection

A multi-sensor approach was adopted to construct a comprehensive time-series analysis of water hyacinth extent:

- **Landsat 8/9 OLI/TIRS:** Landsat 8 and 9 Surface Reflectance Tier 1 Level-2 products (USGS^65^) were acquired for the period 2013–2024, providing an 11-year temporal dataset. Images were filtered for cloud cover ≤ 20% and temporally constrained to October–December each year to capture peak water hyacinth infestation periods coinciding with post-wet season conditions. Surface reflectance products were processed using standard scaling factors to convert digital numbers to surface reflectance values. The datasets were merged to ensure temporal continuity across sensor transitions. These datasets provide analysis-ready imagery at a 30 m spatial resolution.
- **Sentinel-2 MSI**: Sentinel-2 Multispectral Instrument (MSI) Surface Reflectance Harmonized products were utilized for the periods 2016–2024 (NDVI analysis) and 2018–2024 (FAI and combined indices)^43^. Cloud cover filtering was applied with a threshold of ≤ 20% cloudy-pixel percentage. The 10-m spatial resolution of Sentinel-2 provided greater detail than Landsat’s 30-m resolution, enabling more precise delineation of water hyacinth patches.
- **Sentinel-1 C-SAR**: Sentinel-1 Ground Range Detected (GRD) products in Interferometric Wide (IW) mode were acquired for the period 2014–2025. Data were filtered to ensure consistent VH polarization, a filtering approach that proved highly effective for detecting aquatic vegetation. VH polarization data were processed using unit scaling with detection thresholds optimized for floating vegetation signatures. The GRD radar data provided cloud-independent monitoring capabilities, ensuring temporal continuity during periods of persistent cloud cover that commonly affect optical sensors in tropical regions.

The multi-sensor design reflects the staggered availability of satellite platforms over the 11-year study period. Of the 36 monthly observations (October–December 2013–2024), Landsat 8/9 provides the sole optical coverage for 16 months (2013–2017), a period that encompasses the pre-invasion baseline and early build-up phase preceding the peak infestation of 2018–2019. Starting in December 2018, 19 months of concurrent Landsat and Sentinel-2 observations enabled direct cross-sensor comparisons. Sentinel-1 C-SAR radar data are available for 28 of the 36 months (from late 2014 onward), providing cloud-independent temporal continuity. This architecture ensures that the temporal record is not truncated to the Sentinel-2 era alone. At the same time, the environmental coherence framework (Section “Validation approach: environmental coherence framework”) provides a transparent, quantitative basis for evaluating the relative performance of each sensor–index combination.

For all sensors, the time series was constrained to the post-rainy season months of October, November, and December to capture peak water hyacinth proliferation. A cloud-masking procedure was applied to all optical images (Landsat and Sentinel-2) using the quality assessment (QA) bands (QA_PIXEL and QA60, respectively) to remove pixels contaminated by clouds and their shadows. Sentinel-1 images were pre-processed in GEE using a standard workflow that included orbit file application, thermal noise removal, radiometric calibration to Sigma^0^ (σ∘), and terrain correction. A 5 × 5 Lee speckle filter was subsequently applied to reduce granular noise.

### Hydrometeorological datasets

To investigate potential environmental drivers of water hyacinth dynamics, the following datasets were analyzed within the GEE:

- **Multi-source evapotranspiration analysis:** Monthly evapotranspiration rates (mm/day) were derived from the following three complementary datasets to provide robust ET estimates: 1) MODIS Global Terrestrial Evapotranspiration data, with an 8-day temporal resolution and 500 m spatial resolution, and were converted from the original units to daily rates; 2) ERA5-Land Daily Aggregated data, with daily temporal resolution and 11 km spatial resolution, provided comprehensive atmospheric forcings; and 3) FLDAS Noah Land Surface Model data, with monthly temporal resolution and 11 km spatial resolution, offered ground-validated estimates. Data from multiple sources were ensemble-averaged when available to minimize uncertainties and provide robust monthly ET estimates. The purpose of this approach is to mitigate the inherent biases and uncertainties present in any single evapotranspiration (ET) dataset. MODIS is an observation-based product, while ERA5-Land and FLDAS are reanalysis/model-based products. Each has its own strengths, weaknesses, and potential errors. By averaging them, a more robust composite time series is created.
- **Precipitation**: Monthly precipitation totals (mm/month) were extracted from the Climate Hazards Center InfraRed Precipitation with Station (CHIRPS) daily dataset with 5.56 km spatial resolution. Daily precipitation values for the study area were temporally aggregated to monthly totals. CHIRPS data were selected for their demonstrated accuracy in tropical highland regions and extensive indirect validation against ground-based observations.
- **Lake water level**: Monthly lake surface water levels (in meters) were acquired via the Hydroweb API, which provides satellite altimetry data. Lake Tana water levels provide operational measurements of water levels derived from multiple satellite altimetry missions. Monthly composites were generated from available altimetry observations, with statistical indirect validation applied to ensure temporal consistency and data reliability.
- **Temperature and humidity variables**: Detailed meteorological parameters were extracted from the AgERA5 daily dataset, which provides high-resolution agricultural and ecological reanalysis data specifically designed for environmental applications. Data were extracted for October–December of each year from 2013 to 2024, corresponding to the water hyacinth monitoring period.
  - The following air temperature variables at 2-m height were systematically extracted and processed: (1) daily maximum (24-h maximum), (2) daily minimum (24-h minimum), and (3) daily mean (24-h average). For each variable, monthly minimum, maximum, and mean statistics were calculated from all daily observations within each month.
  - The following humidity variables were extracted to characterize atmospheric moisture conditions: 1) Specific humidity (mean specific humidity at 2-m height expressed in kg/kg); 2) Morning relative humidity (relative humidity at 06:00 local time expressed as percentage); and 3) Afternoon relative humidity (relative humidity at 15:00 local time expressed as percentage).

For each meteorological variable, a spatial reduction was performed over the study area using minimum, maximum, and mean reducers. This data was processed employing a 10 km spatial scale to balance computational efficiency with spatial representativeness.

### Water hyacinth mapping and area estimation

To create a consistent, cloud-reduced image for each month of the study period, a compositing method was applied to each satellite image collection. *For the optical datasets (Landsat 8/9 and Sentinel-2)*, a monthly median composite was generated. This technique computes the median value for each pixel across all available images for a given month. This is a robust method for removing clouds, cloud shadows, and other transient artifacts. *For the all-weather Sentinel-1 radar data*, a monthly mosaic was created to ensure complete spatial coverage. The total water hyacinth area was then estimated using a series of spectral indices, radar backscatter analysis, and data fusion techniques applied to these monthly composites.

#### Water hyacinth single-index detection

This study uses water and vegetation indices with pre-determined thresholds developed in previous studies^31^. The following spectral indices—FAI, NDVI, and NDWI—were calculated from the respective monthly composites to differentiate water hyacinth from open water:

1. **The floating algae index (FAI)** was developed to detect cyanobacteria blooms^44^. This index effectively distinguishes floating vegetation from surrounding water by exploiting the spectral signature differences in the near-infrared and short-wave infrared regions. Although the FAI was initially created for MODIS images, it can also be computed using the Landsat 8 reflectance from bands 4, 5, and 6 and from Sentinel-2 bands 4, 8, and 11 using the following linear baseline algorithm:
  1 $$FAI={R}_{rc, B5}-\left[{R}_{rc, B4}+{(R}_{rc, B6}-{R}_{rc, B4}\right)\times (\frac{\left({\lambda }_{B5}-{\lambda }_{B4}\right)}{\left({\lambda }_{B6}-{\lambda }_{B4}\right)})] \text{for Landsat }8/9$$
  2 $$FAI={R}_{rc, B8}-\left[{R}_{rc, B4}+{(R}_{rc, B11}-{R}_{rc, B8}\right)\times (\frac{\left({\lambda }_{B8}-{\lambda }_{B4}\right)}{\left({\lambda }_{B11}-{\lambda }_{B4}\right)})] \text{for Sentinel}-2$$
  where R_rc, Bi_ is the Rayleigh-corrected reflectance for the i^th^ band, and λ_Bi_ is the center wavelength for the i^th^ band.
2. **Normalized difference vegetation index (NDVI)** has been widely used for detecting vegetation areas^45^, for mapping temporal changes in lake areas^46^, and cyanobacterial blooms^47^. NDVI is calculated as follows:
  3 $$NDVI= \frac{{R}_{B5}-{R}_{B4}}{{R}_{B5}+{R}_{B4}} \text{for Landsat }8$$
  4 $$NDVI= \frac{{R}_{B8}-{R}_{B4}}{{R}_{B8}+{R}_{B4}} \text{for Sentinel }2$$
3. N**ormalized difference water index (NDWI)** has been used as a primary tool to enhance water features. For example, McFeeters^48^ proposed an NDWI that combines the green and NIR bands to distinguish between water and non-water areas. Gao^49^ developed an NDWI that combines NIR and SWIR bands to estimate vegetation water content, while Rogers and Kearney^50^ modified Gao’s NDWI by using the red band instead of the NIR band. Xu^51^ found that green and short-wave infrared (SWIR) band combinations could separate built-up features from water features. The general form of the NDWI can be written as:
  5 $${NDWI}_{i,j}= \frac{({R}_{Bi}-{R}_{Bj})}{{(R}_{Bi}+{R}_{Bj})}$$
  The subscripts *Bi* and *Bj* represent the band numbers used for the NDWI calculation.

The single-index detection employed the following sensor-specific optimized thresholds determined through iterative analysis and supported by relevant literature. For the Floating Algae Index (FAI), a threshold of 0.005 was applied for Landsat, aligning with findings from Liu et al.^52^, who reported that thresholds in the range of 0.005–0.07 were effective for identifying floating algae in small eutrophic lakes using Landsat-8 imagery^52^. For Sentinel-2, a lower FAI threshold of 0.002 was used, justified by the higher sensitivity of the MSI sensor and the application of adaptive threshold methods reported by Muzhoffar et al.^53^, which demonstrated reliable macroalgae detection with Sentinel-2 FAI values as low as 0.002–0.01^53^.

For vegetation detection using the Normalized Difference Vegetation Index (NDVI), a uniform threshold of 0.3 was applied to both Landsat and Sentinel-2. This value is widely cited in aquatic vegetation and algal bloom studies, including those by Wang et al.^54^, who effectively used an NDVI threshold of 0.3 to separate vegetated and non-vegetated water surfaces in high-turbidity environments^54^.

#### Water hyacinth combined-index detection

Combined index methods integrate complementary indices using logical intersection operations to minimize false positives and enhance classification accuracy:

- NDVI + FAI: Combined vegetation vigor assessment with floating algae detection.
- NDWI + FAI: Enhanced water-vegetation boundary delineation with floating vegetation identification.
- FAI + NDVI + SWIR: Comprehensive approach incorporating vegetation health, floating material detection, and moisture content assessment.

For each index combination, a thresholding technique was applied to the monthly composite images to generate a binary classification map that distinguished water hyacinth from all other surface types.

- ***Sentinel-2 (paired indices)***
  - NDWI + FAI: FAI > 0.002 and NDWI < 0.0.
  - FAI + NDVI + SWIR: NDVI > 0.30 and FAI > 0.01 and SWIR < 0.10.

- ***Sentinel-2 (composite rule for robustness)***

It utilized a union-of-masks strategy: (NDVI > 0.20 and SWIR < 0.10) OR (NDWI < 0 and FAI > 0.005) OR (NDVI > 0.30 and FAI > 0.01 and SWIR < 0.10).

- ***Landsat 8/9***
  - NDVI + FAI: NDVI > 0.25 and FAI > 0.02.
  - NDWI + FAI: NDWI < 0.0 and FAI > 0.02.

All masks were generated from monthly medians (optical) or mosaics (radar), then clipped to the region of interest.

#### Water hyacinth radar-based detection

Radar-based detection has been used to identify water hyacinth because the water hyacinth mats alter the water surface roughness, resulting in a significant increase in radar backscatter compared to calm open water. Sentinel-1 VH polarization backscatter values, after radiometric calibration to Sigma^0^ (σ°), were converted from decibels (dB) to a linear 0–1 range through unit scaling (i.e., normalizing by the observed minimum and maximum backscatter values within the study area). This transformation is necessary because dB values are logarithmic and not directly suitable for proportional thresholding. The detection threshold of unit-scaled VH > 0.20 was empirically determined by examining the backscatter distribution over known water hyacinth-infested areas in the northeastern littoral zones of Lake Tana versus adjacent open-water areas, using concurrent cloud-free optical imagery as reference. This threshold corresponds to the optimal separation point between the bimodal distribution of vegetated surfaces (higher roughness, higher backscatter) and calm open water (smooth surface, lower backscatter). The radar approach provided consistent monitoring capabilities during cloudy periods when optical sensors were limited.

#### Water hyacinth detection using hybrid optical-radar fusion

Hybrid Optical-Radar Fusion is a novel integration methodology that combines optical and radar data sources using adaptive logic based on atmospheric conditions. This method systematically merges the high-confidence classifications from the optical FAI + NDVI + SWIR method with the radar classification. The primary logic fills areas in the optical classification obscured by clouds with the corresponding classification from the all-weather Sentinel-1 radar data, thus creating a more spatially and temporally complete data layer. For the fusion threshold, VH < 0.10 and (VV/VH) > 1.3 were used. When cloud cover exceeded 30%, radar-only detection was employed. Under clear-sky conditions, optical indices served as the primary indicators, with radar data providing supplementary validation. This approach maximizes temporal coverage while maintaining classification accuracy.

### Areal quantification and temporal analysis

Binary classification masks were converted to area measurements using pixel-based calculations with appropriate spatial scaling for each sensor system. Each detection method operates at the native spatial resolution of its source sensor: 30 m for all Landsat 8/9-based indices (FAI, NDVI, NDVI + FAI, NDWI + FAI), 10 m for all Sentinel-2-based indices (FAI, NDVI, NDVI + FAI, NDWI + FAI, FAI + NDVI + SWIR), 10 m for Sentinel-1 radar (VH polarization), and 10 m for the Hybrid Fusion method. No resampling between resolutions was performed; area estimates reflect the native pixel size of each sensor. The minimum mappable unit is therefore approximately 900 m^2^ for Landsat-based methods and 100 m^2^ for Sentinel-based methods. Isolated water hyacinth patches smaller than these thresholds, or patches that only partially fill a pixel, may be missed or underestimated. However, water hyacinth infestations at Lake Tana typically form dense, contiguous mats spanning hundreds of meters along the northeastern and eastern littoral zones and are well within the detection capability of both sensor systems. Regional statistics were computed by summing across the study area, with spatial resolutions of 30 m for Landsat, 10 m for Sentinel-2, and 10 m for Sentinel-1. Results were converted to square kilometers for consistent reporting and analysis.

Monthly median composites were created to balance reductions in short-term variation with the preservation of seasonal patterns. The October–December analysis window was selected for three reasons: (i) cloud cover during the Ethiopian main rainy season (June–September) routinely exceeds 70–90%, rendering optical satellite imagery unusable for consistent monthly compositing even with aggressive cloud filtering; (ii) water hyacinth proliferation at Lake Tana peaks during the post-rainy season when receding water levels expose nutrient-rich littoral zones under favorable temperature and humidity conditions^3,13^, and (iii) this period provides the most management-relevant information for planning intervention strategies. This temporal scope means that full annual cycle dynamics, including potential residual mat persistence during the rainy season, are not captured, which is acknowledged as a limitation. Year-round radar-based monitoring could partially address this gap in future work.

### Validation approach: environmental coherence framework

A critical methodological consideration in long-term, large-area remote sensing studies is the selection of an appropriate validation strategy. The prevailing literature recognizes a hierarchy of validation approaches for satellite-derived Earth observation products^8^. This hierarchy comprises: (i) direct validation against fiducial reference measurements (e.g., field-collected ground truth), (ii) indirect validation, in which a derived product is evaluated through its correlation with secondary variables for which independent reference data exist, and (iii) inter-product consistency checking. While direct validation against field-collected ground truth represents the ideal scenario, it is frequently impractical for retrospective, multi-decadal, or large-area studies, particularly in data-scarce regions^55,56^. A systematic review of 89 validation studies found that product inter-comparison and indirect validation were the most commonly employed methods (38.5%), and 65.9% of studies used more than one validation method^56^.

In the present study, systematic field-based ground truth collection was not feasible across the full 11-year study period (2013–2024) for several well-documented reasons specific to Lake Tana: (a) retrospective satellite observations cannot be validated against field data that were never collected; (b) the rapid spatial redistribution of water hyacinth mats—where even a one-day gap between satellite overpass and GPS tracking significantly degrades agreement^13^—introduces fundamental temporal mismatch errors,(c) dense water hyacinth mats obstruct boat navigation and pose safety hazards to field teams^57^, and (d) the lake’s ~ 3060 km^2^ surface area renders spatially comprehensive field surveys logistically and financially prohibitive, particularly in the absence of dedicated project funding^4^.

Accordingly, this study adopts an environmental coherence framework as its primary validation approach, following the indirect validation paradigm^8^. The underlying rationale is that if a remote sensing indicator accurately captures water hyacinth dynamics, its time series should exhibit statistically significant correlations with the known environmental drivers of water hyacinth growth—specifically, lake water level, relative humidity, and evapotranspiration—in directions consistent with established ecological understanding. This approach is consistent with validation practices employed in comparable studies of aquatic vegetation and algal bloom dynamics^58–61^, as well as in the Lake Tana water hyacinth literature specifically^3,13^. We acknowledge that environmental coherence does not replace traditional pixel-level accuracy assessment,rather, it provides complementary evidence of ecological validity, particularly for long-term time-series studies in data-scarce tropical regions.

## Results and discussion

Tables 1, 2, and 3 summarize the core outputs of our analysis: (i) indicator-derived estimates of water hyacinth infested area from optical, radar, and hybrid methods are presented in Table 1; (ii) the hydro-meteorological conditions for the study window are presented in Table 2; and (iii) pairwise correlations between each indicator series and the hydro-meteorological variables are provided in Table 3.

**Table 1:** Monthly Water Surface Detection by Sensor/Index at Lake Tana (2013–2024).

No	Year	Month	Cloud Cover (%)		FAI (km^2^)		NDVI (km^2^)		NDVI + FAI (km^2^)		NDWI + FAI (km^2^)		FAI + NDVI + SWIR (km^2^)	Radar (km^2^)	Hybrid Fusion (km^2^)
			Sentinel‑2	Landsat	Landsat	Sentinel‑2	Landsat	Sentinel‑2	Landsat	Sentinel‑2	Landsat	Sentinel‑2	Sentinel‑2	Sentinel‑1	Sentinel‑2
1	2013	10		1.42	2.67		1.69		1.69		1.45				
2	2013	11		7.57	0.05		0.02		0.02		0.03				
3	2013	12		1.33	2.87		1.71		1.71		1.62				
4	2014	10		0.54	0.16		0.05		0.05		0.03				
5	2014	11		3.21	5.45		3.62		3.62		2.70				
6	2014	12		2.04	5.51		4.43		3.28		2.68			5.02	
7	2015	10		7.67	6.39		4.30		4.30		3.18				
8	2015	11		6.19	6.69		4.68		4.68		3.95				
9	2015	12		3.17	6.52		4.42		4.42		4.08				
10	2016	10		10.27	6.50		4.68		4.68		3.94		6.8		
	6.10	4.89													
11	2016	11		0.11	7.26		4.63		4.63		3.73			6.72	5.78
12	2016	12		1.04	8.18		5.71		5.71		4.50			6.85	13.29
13	2017	10												9.12	4.01
14	2017	11		3.08	14.35		8.03		8.03		7.13			13.10	2.92
15	2017	12		0.16	15.39		12.78		12.78		9.83			13.15	20.58
16	2018	10		3.01	13.69		11.66		11.66		10.29			10.58	4.59
17	2018	11		7.67	31.34		16.31		9.53		7.36			13.41	1.5
18	2018	12	0.94	1.25	20.95	15.98	12.39	14.71	12.39	14.71	10.70	13.58	15.20	14.31	4.78
19	2019	10	4.81	10.57	1.23	12.66	0.99	11.48	0.99	11.48	0.80	10.45	12.07	14.54	4.38
20	2019	11	8.59	3.33	26.72	24.13	22.47	15.13	21.26	15.13	18.62	11.86	9.61	26.53	11.18
21	2019	12	4.65	0.68	19.49	19.33	17.78	15.00	17.78	15.00	15.80	13.45	16.16	22.02	18.78
22	2020	10	10.08	13.03	23.32	14.65	66.05	10.61	6.47	10.61	6.53	8.49	10.05	9.94	2.96
23	2020	11	4.20	0.03	15.41	12.83	13.87	11.98	13.87	11.98	12.35	11.35	9.59	15.06	2.43
24	2020	12	3.24	0.37	16.21	12.53	8.92	9.01	8.92	9.01	7.67	7.85	9.24	11.24	5.67
25	2021	10	10.00	10.02	11.62	16.61	10.32	8.18	10.29	8.18	9.87	7.68	9.25	10.45	1.18
26	2021	11	3.88	0.58	25.36	15.68	9.32	9.52	9.32	9.52	6.56	8.51	11.48	9.19	4.34
27	2021	12	1.48	1.85	15.59	12.96	12.99	9.84	12.99	9.84	9.82	8.97	10.33	12.83	5.1
28	2022	10	8.57	8.36	11.44	7.83	8.09	5.74	7.52	5.73	5.70	5.30	6.42	7.71	0.98
29	2022	11	5.00	2.75	10.28	10.15	8.29	7.36	8.21	7.36	6.35	6.38	7.38	10.02	3.94
30	2022	12	6.97	3.96	12.97	9.16	7.32	7.27	7.31	7.27	8.91	6.39	6.41	6.83	4.5
31	2023	10	8.48	7.70	10.20	9.08	8.14	7.05	8.14	7.05	6.83	6.10	7.47	5.14	8.69
32	2023	11	2.86	2.92	10.67	11.28	7.83	6.47	7.83	6.47	5.35	4.97	7.03	6.04	0.71
33	2023	12	3.18	2.18	14.78	14.07	8.73	7.78	8.73	7.78	6.04	6.05	7.16	9.35	14.57
34	2024	10	12.87	9.53	14.73	10.48	11.56	8.24	11.27	8.24	10.15	7.12	8.81	15.42	0.73
35	2024	11	8.98	6.20	20.62	16.43	14.06	14.43	14.04	14.43	11.09	12.15	15.76	16.24	16.45
36	2024	12	2.16	0.14	18.96	15.50	12.17	13.26	12.17	13.26	9.27	11.18	12.42	13.53	0.98

**Table 2:** Monthly Hydroclimate Summary for Lake Tana (2013–2024).

No	Year	Month	Average evapotranspiration (mm/day)	Lake Tana water level (m)	Precipitation (mm/month)	Temperature (°C) — daily Max.			Temperature (°C) — daily Min.			Temperature (°C) — Daily Mean			Relative humidity 06h (%)			Relative humidity 15h (%)		
						Max.	Min.	Mean	Max.	Min.	Mean	Max.	Min.	Mean	Max.	Min.	Mean	Max.	Min.	Mean
1	2013	10	4.42	1,787.08	6,034	24.84	20.18	22.78	19.69	11.23	17.16	21.82	16.84	19.70	97.91	66.21	79.78	82.25	38.12	61.10
2	2013	11	3.41	1,786.47	819	25.59	20.86	23.08	19.75	11.99	17.20	21.80	17.45	20.12	91.92	51.91	71.34	73.55	34.13	55.35
3	2013	12	4.85	1,785.94	82	25.38	20.30	22.77	20.00	8.76	16.36	21.25	15.85	19.41	83.53	42.49	63.34	66.49	22.22	46.76
4	2014	10	4.02	1,787.31	5,173	24.50	21.06	22.63	19.54	12.35	17.20	21.83	17.28	19.77	98.62	63.67	79.51	78.30	39.35	60.61
5	2014	11	4.00	1,786.70	682	25.63	21.54	23.38	19.94	11.54	17.20	21.94	17.68	20.24	83.57	51.28	69.62	66.07	32.03	51.13
6	2014	12	5.38	1,786.18	468	25.79	21.88	23.21	20.27	10.72	16.90	21.65	17.56	19.98	79.38	41.25	63.70	61.30	18.45	46.34
7	2015	10	3.38	1,787.37	3,144	25.98	20.49	24.11	20.66	12.90	18.18	23.00	17.99	21.13	96.78	63.76	77.11	73.98	39.38	56.41
8	2015	11	3.30	1,786.74	1,107	25.95	21.03	23.60	20.49	12.28	17.87	22.53	17.68	20.75	91.81	53.72	70.49	74.29	30.07	54.43
9	2015	12	4.32	1,786.23	470	25.56	21.32	23.19	20.22	10.59	17.48	21.85	16.90	20.17	86.65	48.60	66.43	68.25	31.73	53.68
10	2016	10	3.41	1,787.18	3,845	25.56	21.63	23.52	20.48	12.38	17.99	22.82	17.89	20.74	96.45	60.95	76.87	77.24	36.53	56.80
11	2016	11	3.71	1,786.57	410	26.06	21.58	23.71	20.32	11.06	16.82	21.90	17.54	20.31	80.14	42.72	66.24	61.40	24.09	44.96
12	2016	12	4.33	1,786.05	80	26.62	22.16	23.64	20.21	10.46	16.68	22.14	17.71	20.16	74.49	36.24	61.47	58.62	17.70	43.70
13	2017	10	3.70	1,786.93	5,304	25.64	21.28	23.31	20.52	13.24	18.04	22.25	18.15	20.71	98.47	60.60	77.83	76.46	34.01	60.22
14	2017	11	3.47	1,786.35	665	25.86	21.54	23.73	19.76	11.87	17.43	21.91	18.46	20.64	86.88	48.89	69.19	67.70	29.32	48.96
15	2017	12	4.42	1,785.79	84	26.24	22.07	23.57	19.70	10.15	16.66	21.79	17.77	20.10	76.14	36.94	61.50	55.29	18.00	42.57
16	2018	10	3.46	1,786.77	3,791	25.32	20.58	23.18	20.15	12.10	17.64	22.26	17.44	20.30	99.58	59.45	77.78	76.51	38.06	58.49
17	2018	11	3.87	1,786.16	1,595	25.02	20.15	23.15	20.09	11.33	17.29	21.77	17.11	20.19	90.11	53.15	70.24	70.66	29.15	52.59
18	2018	12	4.72	1,785.64	376	25.94	22.14	23.62	20.60	11.00	17.57	22.10	18.09	20.67	82.95	53.03	64.16	66.18	27.88	49.83
19	2019	10	3.53	1,786.83	6,294	25.25	19.95	23.00	19.74	12.20	17.31	21.73	16.92	19.96	97.42	68.67	79.52	79.69	43.29	60.29
20	2019	11	4.37	1,786.23	1,110	25.57	21.55	23.27	20.42	12.28	17.88	22.07	17.72	20.46	90.65	55.66	72.51	75.68	35.75	57.06
21	2019	12	4.39	1,785.70	819	25.34	21.87	23.35	20.27	10.15	17.34	21.75	17.28	20.31	85.90	48.97	64.49	70.90	26.92	51.20
22	2020	10	3.85	1,787.03	3,124	25.55	21.19	23.16	19.79	12.61	17.55	22.26	17.29	20.40	97.37	57.04	77.59	72.27	34.31	57.25
23	2020	11	2.84	1,786.45	748	26.03	21.79	23.67	20.52	11.80	17.34	22.15	18.49	20.66	85.47	45.99	66.99	66.62	24.06	48.11
24	2020	12	4.31	1,785.93	227	26.44	22.11	23.67	20.39	10.86	17.27	22.11	18.30	20.58	79.72	42.92	63.79	62.07	19.98	46.18
25	2021	10	3.23	1,787.30	3,984	25.18	21.17	23.31	19.96	12.68	17.63	22.08	18.04	20.38	96.16	56.03	77.24	79.31	36.20	57.98
26	2021	11	3.12	1,786.70	1,028	25.70	21.45	23.63	19.91	12.05	17.39	21.96	17.24	20.49	83.23	54.82	69.24	63.62	28.83	50.18
27	2021	12	4.29	1,786.18	305	26.24	21.34	23.75	20.08	11.11	16.94	21.73	17.58	20.19	82.67	41.06	63.77	63.40	18.45	45.18
28	2022	10	3.73	1,787.38	4,693	25.02	20.77	22.90	19.75	12.50	17.45	22.06	17.39	20.06	97.17	56.26	78.37	77.81	36.22	60.14
29	2022	11	3.98	1,786.75	884	25.62	22.09	23.43	19.96	11.34	17.29	21.75	17.95	20.47	82.77	52.02	69.74	61.79	31.70	49.44
30	2022	12	4.54	1,786.23	287	25.21	21.52	22.93	20.10	10.38	17.01	21.41	17.26	19.98	78.60	48.42	62.31	59.10	29.66	48.03
31	2023	10	3.36	1,787.20	5,445	26.24	20.80	24.14	20.83	13.47	18.34	23.41	17.70	21.21	96.04	62.93	76.43	72.87	34.14	56.29
32	2023	11	3.64	1,786.58	2,133	26.06	20.04	23.64	20.41	12.38	17.92	22.67	17.47	20.77	90.96	48.08	70.96	74.92	28.22	53.59
33	2023	12	4.48	1,786.08	82	26.30	22.44	23.78	20.98	11.00	17.75	22.23	17.67	20.77	79.45	46.48	61.81	59.91	28.46	47.30
34	2024	10	3.58	1,786.92	5,974	25.27	20.95	23.02	20.10	13.12	17.76	22.26	17.49	20.21	98.25	69.69	79.99	81.09	45.82	63.36
35	2024	11	3.59	1,786.31	1,300	25.14	20.06	23.21	19.95	11.97	17.52	21.88	17.03	20.41	91.26	54.69	71.54	78.38	35.16	54.61
36	2024	12	4.97	1,785.81	107	25.46	22.16	23.32	19.90	10.09	16.82	21.42	17.05	20.07	81.99	49.59	63.03	62.19	24.82	46.63

**Table 3:** Coefficient matrix by method versus hydroclimate variables (2013–2024).

Method	Average evapotranspiration	Lake Tana water level	Precipitation	Temperature Max.			Temperature Min.			Temperature Mean			Relative humidity 06h			Relative humidity 15h		
				Max.	Min.	Mean	Max.	Min.	Mean	Max.	Min.	Mean	Max.	Min.	Mean	Max.	Min.	Mean
Landsat | FAI	0.02	−0.37	−0.28	0.07	0.20	0.20	0.14	0.00	0.14	−0.03	0.15	0.27	0.24	0.19	0.23	0.23	0.25	0.22
Sentinel2 | FAI	0.23	−0.44	−0.35	−0.09	0.22	0.00	0.08	−0.19	0.03	−0.22	−0.02	0.01	0.45	0.52	0.44	0.60	0.49	0.52
Landsat | NDVI	−0.01	−0.04	−0.03	0.01	0.10	0.01	−0.05	0.15	0.14	0.07	0.05	0.15	0.00	0.01	−0.07	0.06	0.02	0.02
Sentinel2 | NDVI	0.29	−0.61	−0.34	−0.14	0.17	−0.07	0.00	−0.34	−0.19	−0.34	−0.10	−0.11	0.44	0.57	0.40	0.56	0.57	0.54
Landsat | NDVI + FAI	0.03	−0.41	−0.26	0.15	0.30	0.27	0.26	−0.02	0.18	0.02	0.30	0.30	0.11	0.17	0.17	0.09	0.21	0.14
Sentinel2 | NDVI + FAI	0.29	−0.61	−0.34	−0.13	0.17	−0.07	0.00	−0.34	−0.19	−0.34	−0.10	−0.11	0.44	0.57	0.40	0.56	0.57	0.54
Landsat | NDWI + FAI	0.03	−0.38	−0.23	0.09	0.30	0.20	0.24	−0.01	0.18	0.00	0.30	0.27	0.05	0.14	0.12	0.03	0.18	0.10
Sentinel2 | NDWI + FAI	0.23	−0.59	−0.31	−0.14	0.18	−0.06	−0.02	−0.36	−0.26	−0.37	−0.04	−0.14	0.37	0.48	0.35	0.46	0.48	0.46
Sentinel2 | FAI + NDVI + SWIR	0.17	−0.52	−0.22	−0.20	0.02	−0.05	−0.15	−0.33	−0.24	−0.33	−0.25	−0.14	0.29	0.40	0.24	0.44	0.41	0.41
Sentinel1 | Radar	0.08	−0.40	−0.13	−0.26	0.01	−0.21	−0.12	−0.10	0.03	−0.31	−0.05	−0.12	0.35	0.44	0.34	0.40	0.42	0.40
Sentinel2 | Hybrid Fusion-Radar + Optical-Fusion-FAI + NDVI + SWIR	0.35	−0.49	−0.36	0.30	0.29	0.23	0.15	−0.42	−0.19	−0.06	−0.09	0.00	0.07	0.14	0.15	0.18	0.25	0.14
Max	0.35	−0.04	−0.03	0.30	0.30	0.27	0.26	0.15	0.18	0.07	0.30	0.30	0.45	0.57	0.44	0.60	0.57	0.54
Min	−0.01	−0.61	−0.36	−0.26	0.01	−0.21	−0.15	−0.42	−0.26	−0.37	−0.25	−0.14	0.00	0.01	−0.07	0.03	0.02	0.02
Max Abs	0.35	0.61	0.36	0.30	0.30	0.27	0.26	0.42	0.26	0.37	0.30	0.30	0.45	0.57	0.44	0.60	0.57	0.54

The inter-method variation in estimated water hyacinth area visible in Table 1 is expected and reflects three systematic factors: (i) spatial resolution differences, with Landsat’s 30 m pixels producing greater mixed-pixel effects than Sentinel-2’s 10 m pixels; (ii) spectral sensitivity differences, as FAI targets floating-material reflectance, NDVI measures vegetation vigor, and radar detects surface roughness—each capturing a different physical aspect of the same phenomenon; and (iii) threshold stringency, where combined indices using intersection logic (e.g., NDVI > threshold AND FAI > threshold) yield smaller but more conservative area estimates than single indices. This variation across methods is the primary motivation for the environmental coherence framework, which ranks methods by the consistency of their temporal signals with known environmental drivers rather than by absolute area values.

The proliferation of water hyacinth is closely tied to key environmental drivers, including evapotranspiration, water level, temperature, precipitation, and relative humidity. As discussed previously in section “Water Hyacinth in Lake Tana” (“Remote Sensing of Water Hyacinth”), the following studies have established empirical relationships between these variables in Lake Tana and similar tropical lake systems: Evapotranspiration (ET)^41^, Lake level^3^, Precipitation^42^, Temperature (Max, Min, Mean)^42^, and Relative humidity (RH)^41^. These previous studies support ranking the indicators not only by their remote-sensing accuracy but by how closely each correlates with the drivers of water hyacinth growth.

The correlation analysis of environmental variables with remote sensing indicators reveals significant relationships that align strongly with the published literature cited above on water hyacinth ecology and growth patterns. These correlations provide critical indirect validation of the performance of these indicators and support the ranking of detection methods by environmental coherence.

Ranking methodology focusing on environmental correlations using three Environmental Criteria (equal weighting of 33.3% for each):

1. ***Lake Level Correlation***: Absolute correlation with water level.
2. ***Humidity Correlation***: Maximum correlation with relative humidity.
3. ***Environmental Consistency***: Composite metric representing the average strength of an indicator’s correlations with three key environmental drivers:
  - Lake Water Level Correlation (absolute value),
  - Maximum Relative Humidity Correlation (morning 06 h or afternoon 15 h),
  - Evapotranspiration Correlation (absolute value).

As detailed in Section “Validation approach: environmental coherence framework”, the environmental coherence framework adopted here follows the indirect validation paradigm established by Loew et al.^8^ and Gruber et al.^55^. While this approach does not replace traditional field-based accuracy assessment, it offers several complementary strengths that are particularly relevant to long-term, large-area monitoring in data-scarce regions: (a) it enables retrospective evaluation across the full 11-year study period, for which no concurrent field data exist, (b) it provides an ecologically grounded criterion for ranking detection methods based on their sensitivity to established environmental drivers rather than to a single spatial snapshot; (c) it yields temporally independent results that are not contingent on the availability of historical ground truth from a specific date or location; and (d) it is directly transferable to other study sites where similar environmental driver data are available through global reanalysis products, without requiring site-specific field campaigns. Comparable indirect validation approaches have been employed in recent Lake Tana water hyacinth studies^3,13^ and in broader aquatic remote sensing research^58–60,62^.

Table 4 presents the complete relative ranking of the indicators for each evaluation criterion, along with the composite Environmental Score, computed as the equally weighted mean of three sub-criteria—lake level correlation, humidity correlation, and environmental consistency—each normalized to the [0–1] range across the eleven tested indicators. A score of 1.00 indicates that the indicator achieved the highest normalized value across all sub-criteria relative to the other methods; *it does not imply perfect ecological representation in an absolute sense*. The top five indicators, in descending order, are:

**Table 4:** Complete Final Relative Ranking Table.

Rank	Indicator	Lake level correlation	Humidity correlation	Environmental consistency	Environmental score
1	Sentinel-2 NDVI + FAI	−0.61	0.54	0.48	1.00
2	Sentinel-2 NDVI	−0.61	0.54	0.48	1.00
3	Sentinel-2 NDWI + FAI	−0.59	0.46	0.43	0.90
4	Sentinel-2 FAI	−0.45	0.52	0.40	0.84
5	Sentinel-2 FAI + NDVI + SWIR	−0.52	0.41	0.37	0.79
6	Sentinel-1 Radar	−0.40	0.40	0.29	0.67
7	Sentinel-2 Hybrid Fusion	−0.49	0.15	0.33	0.59
8	Landsat FAI	−0.37	0.23	0.21	0.49
9	Landsat NDVI + FAI	−0.41	0.17	0.20	0.47
10	Landsat NDWI + FAI	−0.38	0.12	0.18	0.41
11	Landsat NDVI	−0.04	0.07	0.04	0.09

1. ***Sentinel-2 NDVI + FAI*** (Environmental Score: 1.00—Highest *Relative* Environmental Coherence).
  Strongest lake level correlation (r = −0.61)—optimal drawdown sensitivity
  Maximum humidity correlation (r = 0.54)—optimal growth condition detection
  Highest ET correlation (r = 0.29)—superior hydrological impact capture
  Environmental consistency score: 0.48—best integrated ecological response
  Highest relative environmental coherence across all tested ecological drivers
2. ***Sentinel-2 NDVI*** (Environmental Score: 1.00—Highest *Relative* Environmental Coherence).
  Identical environmental performance to the NDVI + FAI combination
  Strongest correlation with all known environmental drivers
  Demonstrates that NDVI alone captures the complete vegetation signal
  Simple to implement with equivalent ecological validity
3. ***Sentinel-2 FAI*** (Environmental Score: 0.84—Strong Environmental Coherence)
  Moderate lake level correlation (r = -0.44)—good but not optimal drawdown sensitivity
  Strong humidity correlation (r = 0.52)—excellent growth condition detection
  Moderate environmental consistency—solid but not perfect ecological response
  18.9% lower environmental performance than optimal NDVI methods
4. ***Sentinel-1 Radar*** (Environmental Score: 0.67—Moderate Environmental Coherence)
  Moderate environmental correlations reflecting surface roughness changes
  Cloud-independent monitoring capability ensuring temporal continuity
  Useful for gap-filling but not primary ecological detection

The lower environmental coherence of radar relative to optical methods reflects its indirect detection mechanism—surface roughness changes rather than spectral vegetation properties—and does not diminish its operational value. Radar’s primary contribution is to ensure temporal continuity under persistent cloud cover, complementing higher-accuracy optical methods rather than competing with them. This complementary role is operationalized through the Hybrid Optical–Radar Fusion method (Section "Water hyacinth detection using hybrid optical-radar fusion").

4. ***Landsat FAI*** (Environmental Score: 0.49—Weakest Environmental Coherence)
  Weakest environmental correlations across all ecological validation criteria
  Longest temporal record (2013–2024) providing crucial trend analysis
  Coarser spatial resolution limits environmental response detection

**Fig. 3:**
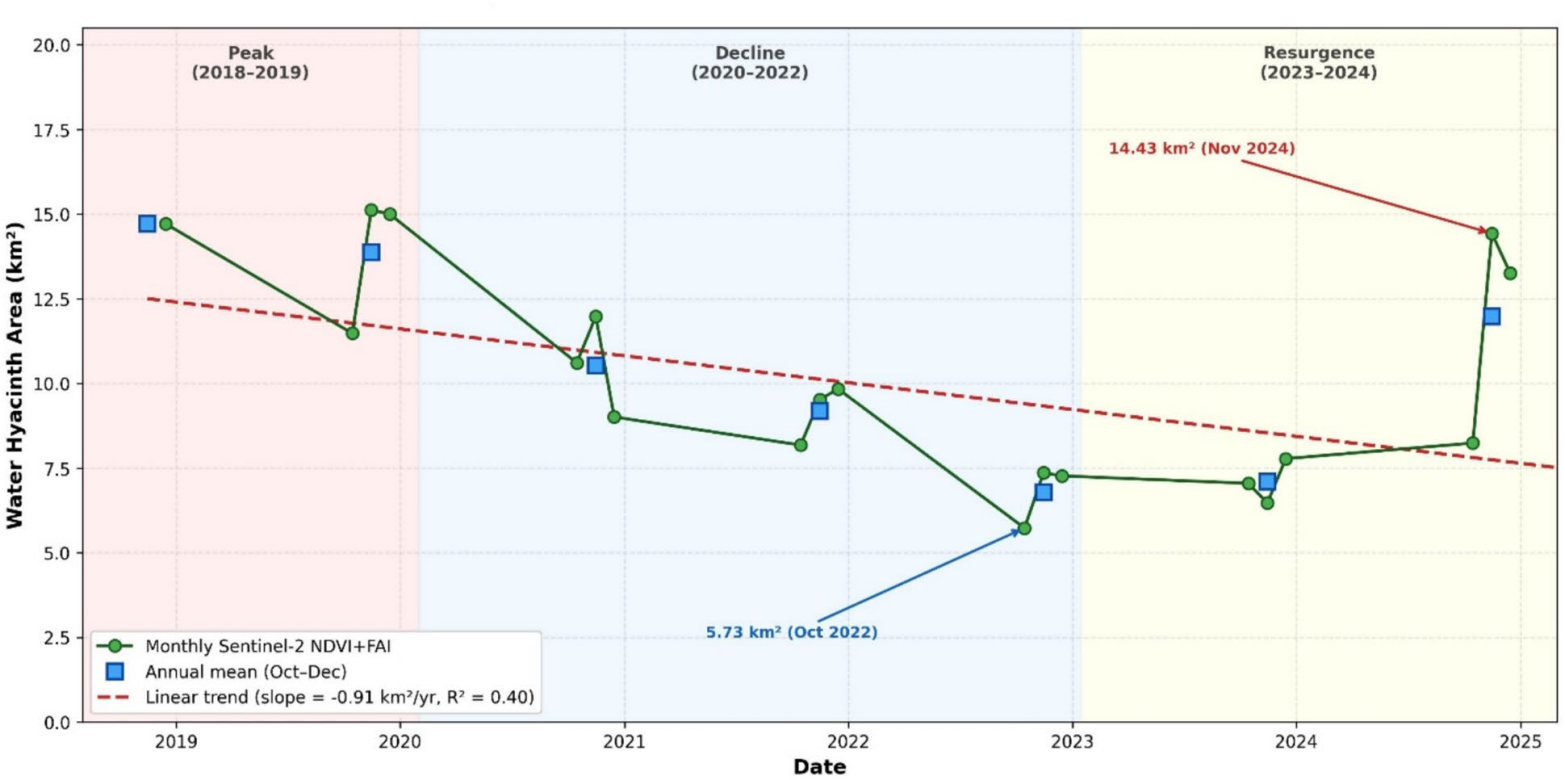
Time series of Water hyacinth areal extent at Lake Tana (Sentinel-2 NDVI + FAI, October-December 2018–2024).

A detailed temporal analysis of water hyacinth infestation, derived from the Sentinel-2 NDVI + FAI composite index, reveals a complex, highly variable pattern from 2018 to 2024 (Sentinel-2 images began in 2018). While a linear regression of the entire dataset indicates a statistically moderate declining trend with an average annual decrease of 0.91 km^2^ (R^2^ = 0.40), this overall trajectory masks significant inter-annual fluctuations that are better understood as distinct ecological phases (Fig. 3). The period begins with a Peak Phase (2018–2019), where the mean water hyacinth areal coverage remained high, consistently exceeding 13 km^2^. Figure 2 shows the maximum area infected with water hyacinth in Lake Tana in December 2019. This was followed by a pronounced decline phase from 2020 to 2022, during which there was a consistent year-over-year reduction in infestation, culminating in the lowest recorded mean annual water hyacinth coverage of 6.8 km^2^ (Sentinel-2 NDVI + FAI) in 2022 (Oct, Nov, and Dec)—a 54% decrease from the 2018 peak. However, this period of remission was not sustained. A recovery phase began in 2023 and intensified dramatically in 2024, with a 68.7% increase in the mean annual water hyacinth coverage in 2024 from the 2022 minimum. This "boom-bust" cycle, characterized by periods of high growth, decline, and then resurgence again, underscores that while a long-term downward trend in the area infested by water hyacinth is present, the lake’s ecosystem remains highly susceptible to rapid re-infestation events, such as the anomalous spike to 14.43 km^2^ observed in November 2024, and highlighted in Table 1 for NDVI + FAI using Sentinel-2.

The intra-annual dynamics exhibit a strong, consistent seasonal pattern, with water hyacinth coverage systematically progressing through a post-rainy-season growth cycle from October to December. Each October from 2019 to 2024 consistently represents the establishment or "pre-peak" phase, with the lowest average water hyacinth coverage of 8.6 km^2^ (Sentinel-2 NDVI + FAI), making it a critical window for management interventions. A rapid expansion phase followed each November from 2019 to 2024, during which water hyacinth coverage increased by an average of 26.5% to 10.8 km^2^. The infestation reached its seasonal average maximum each December from 2018 to 2024, with an average water hyacinth coverage of 10.98 km^2^. Growth appears to plateau in December, suggesting the saturation of suitable habitats or the onset of environmental limiting factors. An analysis of the sources of variability indicates that while the long-term temporal trend accounts for approximately 40% of the variance, the seasonal component contributes about 20%. The remaining 40% is attributable to random environmental factors, such as climatic anomalies, lake-level fluctuations, and potential management actions. This highlights that the predictable seasonal pulse is superimposed on a highly variable inter-annual trend, emphasizing that accurate interpretation and forecasting require integrating hydro-meteorological data to explain the significant year-to-year differences in infestation severity.

## Conclusions

This study demonstrates that a multi-sensor, index-based workflow implemented entirely in Google Earth Engine can deliver robust, management-ready time-series estimates of the extent of water hyacinth in Lake Tana. By integrating Landsat 8/9, Sentinel-2, and Sentinel-1 with targeted indices (FAI, NDVI, and NDWI) and an adaptive optical–radar fusion rule, the workflow produced consistent monthly areal estimates (October–December) while mitigating cloud-induced gaps and preserving spatial detail at 10–30 m resolutions. In parallel, a novel environmental coherence framework—anchored in correlations with lake water level, relative humidity, and evapotranspiration—was developed to systematically evaluate 11 remote-sensing algorithms. The framework was applied to Lake Tana, identifying Sentinel-2 NDVI and NDVI + FAI as the most accurate indicators of water hyacinth infestation. These detectors yielded the highest relative environmental coherence (Relative Environmental Score = 1.00) among the eleven indicators that were evaluated and showed the strongest correlations with established environmental drivers of water hyacinth proliferation.

A notable finding is the substantial performance gap between Sentinel-2 and Landsat indicators: Sentinel-2 NDVI and NDVI + FAI achieve Relative Environmental Scores of 1.00, while Landsat FAI achieves 0.49 and Landsat NDVI achieves 0.09 (Table 4). This gap is attributable to well-documented differences in spatial resolution and spectral design. Landsat 8/9 OLI operates at 30 m resolution, where each pixel covers 900 m^2^—nine times the area of a 10 m Sentinel-2 MSI pixel (100 m^2^). Water hyacinth mats at Lake Tana form spatially heterogeneous, fragmented patches concentrated along shorelines and in shallow bays^3^, where 30 m pixels frequently contain sub-pixel mixtures of water hyacinth, open water, and shoreline vegetation. This mixed-pixel effect attenuates the spectral signal and reduces correlation with environmental drivers, an effect well-documented in cross-sensor comparisons^63,64^. The particularly low Landsat NDVI score (0.09) indicates that broadband NDVI at 30 m resolution is largely insensitive to floating aquatic vegetation in this mixed land–water environment—consistent with the known limitations of coarse-resolution NDVI for aquatic macrophyte detection^4^. Importantly, during the 19-month overlap period (2018–2024), Landsat and Sentinel-2 time series track qualitatively consistent temporal trends (Table 1), both capturing the same boom-bust invasion trajectory. This cross-sensor temporal consistency provides independent corroboration that the underlying invasion dynamics are reliably captured even at coarser resolution, although with reduced precision in absolute areal estimates. The coherence framework’s ability to quantify these inter-sensor differences is itself a methodological contribution, enabling users to make informed sensor selection decisions for operational monitoring.

The superior performance of Sentinel-2 indicators is attributable to the sensor’s higher spatial resolution (10 m vs. 30 m for Landsat 8/9) and narrower spectral bands, which enable more precise delineation of boundaries in spatially heterogeneous floating vegetation^63,64^. Landsat-derived areal estimates for the pre-Sentinel-2 period (2013–2017) consequently carry greater uncertainty than Sentinel-2 estimates, and users of this workflow are recommended to prefer Sentinel-2 indices where temporal coverage permits. Nevertheless, Landsat data remains essential for reconstructing the full 11-year invasion trajectory, including the critical build-up phase that preceded the peak infestation. The NDVI exhibits robust performance, highlighting the critical role of vegetation intensity signals in high-turbidity, nutrient-rich lacustrine environments. This multi-index approach effectively leveraged the complementary spectral signatures of water hyacinth, with FAI capturing the characteristics of floating material, NDVI assessing vegetation vigor, and SWIR providing information on moisture content. The all-weather capability of radar proved particularly valuable during the study period, as it maintained temporal continuity when optical sensors were limited by cloud cover. The Sentinel-1 C-SAR VH polarization effectively distinguished the increased surface roughness caused by water hyacinth mats from that of smooth open-water surfaces. The novel Hybrid Optical-Radar Fusion method (Sentinel-2 FAI + NDVI + SWIR combined with Sentinel-1 radar) showed high variability. While this approach provided comprehensive spatial coverage by filling cloud-obscured gaps in optical data with radar observations, integrating different sensing modalities introduced additional complexity, affecting consistency.

The effectiveness of optical methods despite cloud contamination is attributable to the monthly median compositing approach: by computing the pixel-wise median from all available images within each month, transient clouds are statistically filtered. Within the October–December study window, residual cloud cover after QA-band masking is moderate (typically 1–13% for Sentinel-2; Table 1), providing sufficient cloud-free observations for robust composites. The comparatively lower performance of the Hybrid Fusion method (Environmental Score = 0.59) is explained by the inherent difficulty of merging two sensing modalities that detect different physical properties: optical indices respond to spectral vegetation signatures, while radar responds to surface roughness. The adaptive switching logic—transitioning between optical-primary and radar-only modes at a 30% cloud cover threshold—introduces discontinuities, and the union of two independently calibrated classifications amplifies noise rather than reducing it. The fusion method’s value, therefore, lies in operational gap-filling during heavy cloud cover, not in improved detection accuracy under clear-sky conditions.

The findings reveal a complex invasion trajectory comprising distinct ecological phases: an initial peak (2018–2019), followed by a substantial decline (2020–2022), and a recent resurgence (2023–2024). Despite an overall decline of 0.91 km^2^ per year, the ecosystem remains highly vulnerable to rapid re-infestation, as evidenced by a 68.7% increase in water hyacinth coverage from 2022 to 2024. The consistent seasonal pattern, with October presenting the lowest water hyacinth coverage (8.6 km^2^) and progressive expansion of the infested area through December, provides critical temporal windows for management interventions. The documented invasion dynamics, combined with the accessible GEE-based methodology, enable proactive management strategies and support evidence-based policy decisions for protecting this critical freshwater ecosystem that sustains over 123 million people.

The strong correlations shown in Table 3 between the water hyacinth infested area and environmental variables—particularly lake level (r = −0.61), relative humidity (r = 0.54), and evapotranspiration (r = 0.29)—validate our remote sensing approach and underscore the importance of considering hydro-meteorological conditions in forecasting water hyacinth invasions. The successful implementation of our hybrid optical-radar fusion methodology ensures continuous monitoring capability even during persistent cloud cover, addressing a major limitation in the surveillance of tropical aquatic ecosystems.

This research contributes several methodological advances: (1) establishes a pure environmental validation framework independent of potentially biased historical estimates, (2) demonstrates NDVI’s effectiveness as a standalone indicator for water hyacinth detection, simplifying operational monitoring, and (3) develops a cloud-computing workflow in Google Earth Engine that can be readily adapted to evaluate the extent of water hyacinth in other water bodies.

While this study employs an environmental coherence framework for indirect validation rather than traditional field-based accuracy assessment, the ecological consistency of the results across multiple independent drivers supports the reliability of the sensor rankings. Future work should incorporate targeted field campaigns coordinated with satellite overpasses to provide complementary pixel-level accuracy metrics. Future research should also focus on integrating these remote sensing products with hydrodynamic models to predict the spread patterns of water hyacinth invasions. Validation of the environmental coherence framework on additional water bodies, particularly tropical lakes and reservoirs with documented water hyacinth infestations, could be a priority for future work, and is facilitated by the open-source GEE code and globally available input datasets provided with this study. This information can then be used to develop early warning systems based on the identified environmental thresholds. The established baseline and methodological framework presented here provide the foundation for adaptive management strategies essential for preserving Lake Tana’s ecological integrity and socio-economic value.

## Data Availability

The Google Earth Engine code developed for this study is openly available at 10.5281/zenodo.17563763. The remote sensing datasets used in this study (Landsat 8/9, Sentinel-2, Sentinel-1) are publicly available through Google Earth Engine. Hydro-meteorological data (water levels, temperature, precipitation, evapotranspiration) were obtained from publicly available sources, including NASA POWER, CHIRPS, and TerraClimate datasets accessible through Google Earth Engine. The processed time-series data, statistical analysis results in Excel format, and shapefile input data used for information extraction are all available at the Zenodo repository link provided above.

## Code availability

All custom code developed for this study is publicly available on GitHub at https://doi.org/10.5281/zenodo.17563763. The repository includes Python scripts for all detection algorithms (FAI, NDVI, NDVI + FAI, NDWI + FAI, FAI + NDVI + SWIR, Sentinel-1 radar, and hybrid fusion), environmental data extraction (evapotranspiration, precipitation, water levels, temperature, and humidity), and data processing workflows implemented in Google Earth Engine. The code is provided under an open-source license to facilitate reproducibility and enable adaptation for other aquatic invasive species monitoring applications.
